# Patterns of West Nile Virus in the Northeastern United States Using Negative Binomial and Mechanistic Trait‐Based Models

**DOI:** 10.1029/2022GH000747

**Published:** 2023-04-04

**Authors:** Alexander C. Keyel

**Affiliations:** ^1^ Division of Infectious Diseases Wadsworth Center New York State Department of Health Albany NY USA; ^2^ Department of Atmospheric and Environmental Sciences University at Albany SUNY Albany NY USA

**Keywords:** Arbovirus, vector‐borne disease, negative binomial, temperature‐trait model, climate change, public health

## Abstract

West Nile virus (WNV) primarily infects birds and mosquitoes but has also caused over 2,000 human deaths, and >50,000 reported human cases in the United States. Expected numbers of WNV neuroinvasive cases for the present were described for the Northeastern United States, using a negative binomial model. Changes in temperature‐based suitability for WNV due to climate change were examined for the next decade using a temperature‐trait model. WNV suitability was generally expected to increase over the next decade due to changes in temperature, but the changes in suitability were generally small. Many, but not all, populous counties in the northeast are already near peak suitability. Several years in a row of low case numbers is consistent with a negative binomial, and should not be interpreted as a change in disease dynamics. Public health budgets need to be prepared for the expected infrequent years with higher‐than‐average cases. Low‐population counties that have not yet had a case are expected to have similar probabilities of having a new case as nearby low‐population counties with cases, as these absences are consistent with a single statistical distribution and random chance.

## Key Points

- Small increases in temperature‐based suitability for West Nile virus (WNV) are expected over most of the northeastern US in the next decade
- Years with high and low cases are consistent with the same underlying statistical distribution
- Temperature‐based suitability for WNV in some of the most populous counties is expected to decrease under climate change

## 1 Introduction

**Figure 1:**
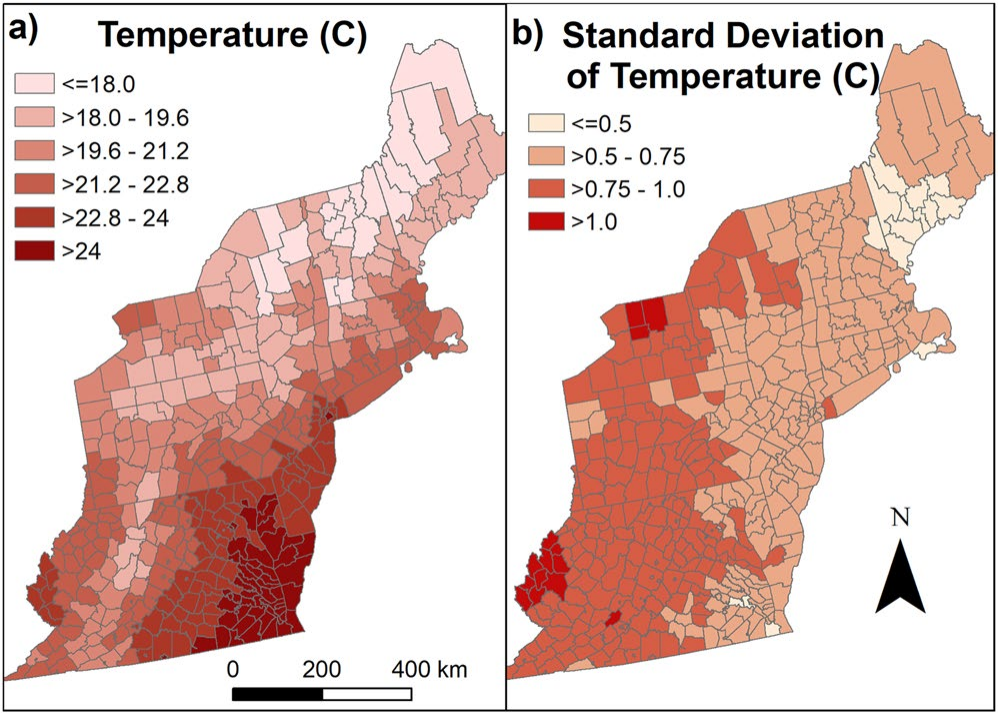
(a) Present‐day mean temperature for July–September, (b) standard deviation of present‐day temperature. Map base layer from 2017 TIGER/Shapefiles (US Census Bureau, 2021).

Climate change is predicted to adversely affect human health and economic productivity (USGCRP, 2018, p. 18). One way climate change is expected to affect human health is through changes to patterns of infectious disease (e.g., Ryan et al., 2015). West Nile virus (WNV) is a vector‐borne disease of public health concern (Hayes et al., 2005; Keyel, Gorris, et al., 2021) and is expected to change its distribution due to climate change (Chen et al., 2013; Hoover & Barker, 2016; Keyel, Raghavendra, et al., 2021). Broadly, WNV is expected to shift northward, but regional temperature‐based analyses show that changes may vary depending on regional differences in temperature (Keyel, Raghavendra, et al., 2021; Morin & Comrie, 2013). For most of the Northeast, temperatures are predicted to warm, especially minimum (night‐time) temperatures (Liu et al., 2017). Precipitation is also predicted to increase, especially in winter, due in part to an increased number of storms (Lynch et al., 2016; Thibeault & Seth, 2014). Summers may see increased run‐off and periods of dryness (Lynch et al., 2016). In the next 10 years, the climate is expected to warm by 0.2–0.5°C (Liu et al., 2017). These predicted changes are within the range of variation in temperature currently experienced (Figure 1).

Probabilistic null models have been previously developed for the United States (Keyel & Kilpatrick, 2021). These models consider a range of possible outcomes, rather than predicting one single number of cases for the future. In one instance, a model that worked well in a non‐probabilistic context (e.g., Keyel et al., 2019) was not able to outperform a probabilistic negative binomial null model in a predictive context. A negative binomial was also shown to be among the best models in a national WNV forecasting challenge (Holcomb et al., 2023). As a consequence, current models for the Northeastern US are very good at describing the range of possible outcomes, but do not provide much information on where in the range of outcomes a particular year will fall.

Mosquito‐temperature‐trait models have been productively used to understand the potential for vector‐borne diseases to respond to climate change (Mordecai et al., 2019; Ryan et al., 2015). They were recently adapted to WNV (Shocket et al., 2020). Statistical models trained on human cases that included an adequate temperature range largely supported the results of mosquito‐trait‐based models for WNV in New York and Connecticut (Keyel, Raghavendra, et al., 2021).

These two approaches serve as complementary measures of WNV risk. One describes the probability distribution for numbers of human neuroinvasive cases, while the other examines the suitability of temperatures for WNV and a key vector species. These measures were used to describe present day risk and examine the potential for present day risks to change in the next decade due to climate change. These measures of risk provide insights into short‐term adaptation measures that can be taken.

## 2 Methods

### 2.1 Negative Binomial Model

Negative binomial model predictions were taken from Keyel and Kilpatrick (2021), as that was found to be among the strongest null models in the Northeast. The negative binomial was chosen instead of the historical null model due to the long time series, and the capacity to downscale model results based on population (Klenke, 2008). The model used here was implemented in R (R Core Team, 2017). This distribution allowed us to cleanly calculate probability of an arbitrary number of human neuroinvasive cases for an arbitrary number of years into the future. One downside of the negative binomial model is it does not predict whether a particular year will have cases or not, but it can give insights into the overall probability of a given number of WNV neuroinvasive cases.

A negative binomial was fit to each county individually, and to groups of multiple counties (Figure S1). Group assignment was subjective and followed the following guidelines: Each group needed to be contiguous with surrounding counties, contain counties from only one state, and include a minimum of 6,00,000 people in each group. No upper group size was imposed, but groups with more than 1,200,000 were examined to see if they could be split into two or more groups. Secondarily, counties with similar population density were preferentially grouped together. The probability a county would have zero WNV cases by chance was calculated, assuming the down‐scaled group negative binomial distribution was true. Future research could examine alternative spatial methods for aggregating counties in order to improve risk estimates.

### 2.2 Climate Change Predictions

This manuscript builds upon this prior work by expanding the use of the mosquito temperature‐trait models to the entire northeast. Briefly, these models use mosquito life history traits to estimate a relative temperature‐based WNV suitability (relative *R*_0_). Equation 1 incorporates traits related to WNV incubation PDR(*T*), dissemination and transmission v*c*(*T*), and with traits related to mosquito life history relating to frequency of biting *a*(*T*), daily mortality rate, *u*(*T*), egg production per female per gonotrophic cycle, EFGC(*T*), egg viability, EV(*T*), larval survival, pLA(*T*), and mosquito development rate MDR(*T*). The virus‐related terms of the equation relate to how fast and effectively the virus can be transmitted, while the mosquito‐related terms define how fast the mosquito population is capable of growing. Traits are combined in a multiplicative framework (see Equation 1, modified from Shocket et al. (2020), see (Keyel, Raghavendra, et al., 2021) for more details) to give *R*_0_(*T*). *R*_0_(*T*) is then scaled to give an index of relative suitability between 0 and 1.

(1) $$R_{0}\left(T\right)=\sqrt{{a\left(T\right)}^{3}*vc\left(T\right)*e^{\frac{-u\left(T\right)}{PDR\left(T\right)}}*EFGC\left(T\right)*EV\left(T\right)*pLA\left(T\right)*MDR\left(T\right)*{u\left(T\right)}^{-3}}$$

Host density and disease duration were assumed to be constant and were omitted from the equation because they cancel out during the index scaling (but see Kilpatrick et al., 2006 for the potential for host heterogeneity to affect these results). Critically, these models look at the contribution of temperature only to risk, other important factors such as breeding habitat availability, land cover (Bradley et al., 2008), or precipitation are not considered (Shocket et al., 2020). These models were developed in the context of *Culex pipiens* (Shocket et al., 2020). *Cx*. *pipiens* is one of the most important mosquito vectors for WNV across the Northeastern US (Andreadis, 2012; Kilpatrick et al., 2005; Simpson et al., 2012; Turell et al., 2005). *Cx*. *pipiens* is closely related to, and hybridizes with *Cx*. *quinquefasciatus* (Farajollahi et al., 2011), an important WNV vector in the Southeastern US (Godsey et al., 2005). Relative risk estimates for *Cx*. *pipiens* and *Cx*. *quinquefasciatus* are similar (see Shocket et al., 2020, Figure 7), especially with respect to the optimum and upper end of the curve. Consequently, these methods are expected to remain valid even if the distributions of these two species shift in the future. Present day conditions and risk expected for 0.5°C warming were examined. Present‐day July–September mean temperatures were derived from GridMET (Abatzoglou, 2013), using the GridMET downloader tool (Wimberly & Davis, 2019) and averaged over the entire period. Model results up to 4°C warming were generated (see Table S2 in Keyel, 2022a). Up to 4°C warming for the 5 most densely populated counties in the Northeast was also examined (Table 1). Warming of up to 4°C is within the realm of possible temperature changes for the Northeast by the end of the century (IPCC, 2014; Liu et al., 2017). This is also the rationale for 0.5°C for an upper‐bound for the increase in temperature in the next decade (4°C/8 decades = 0.5°C per decade). Note that the analyses here calculated trait‐based risk on mean temperatures for the region. Due to the non‐linear response curve, the quantitative results would have differed if relative risk were calculated first, and then averaged. A second source of error for this approach is microclimatic variability, which can affect disease risk (Haider et al., 2017). Third, timing of mosquito activity may allow mosquitoes to buffer against extreme heat and cold (Danforth et al., 2016), thus there is a mismatch between temperature means at a weather station and the temperatures experienced by the mosquitoes. Future research could refine these results, but the broad patterns are expected to be qualitatively similar.

**Table 1:** Relative *R*_0_'s for 5 Major Northeastern US Metropolitan Counties Based on Different Levels of Warming (in C)

County	+0.0	+0.5	+1.0	+1.5	+2.0	+2.5	+3.0	+3.5	+4.0
Suffolk (Boston)	0.88	0.92	0.96	0.99	1.00	1.00	0.98	0.96	0.92
New York (Manhattan)	1.00	1.00	0.98	0.95	0.91	0.86	0.80	0.73	0.66
Philadelphia	1.00	1.00	0.99	0.96	0.92	0.88	0.82	0.75	0.68
Baltimore City	0.98	0.95	0.91	0.86	0.80	0.74	0.66	0.58	0.50
District of Columbia	1.00	0.99	0.96	0.92	0.87	0.82	0.75	0.67	0.597

## 3 Results

**Figure 2:**
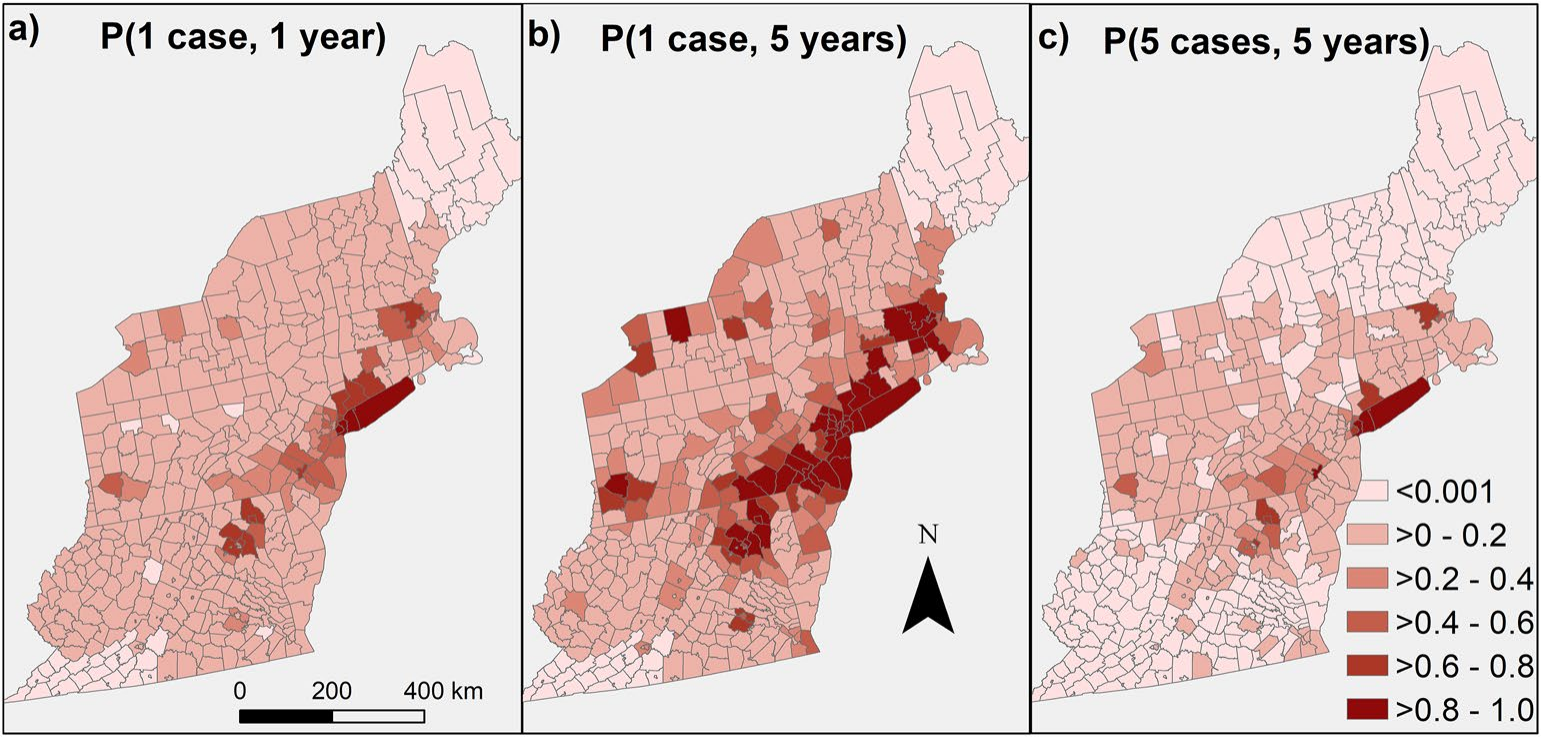
(a) Probability of having one case of West Nile virus (WNV) in the next year, (b) probability of having at least one case of WNV in the next 5 years, and (c) probability of having 1 year with at least 5 cases in the next 5 years. The negative binomial model was fit based on historical cases. Counties with less than 600,000 people were merged with other contiguous counties in the same state until at least a 600,000 person threshold was reached, to ensure a sufficient population size to detect WNV at low incidence. Results were then downscaled back to the county level. Underlying spatial data from 2017 TIGER/Shapefiles from US Census bureau (US Census Bureau, 2021).

Most counties (374 of 433) in the study region had a low probability (<20% chance) of having a single WNV neuroinvasive case in the next year (Figure 2a). Greater than three times as many counties had a high probability of having at least one neuroinvasive case (>60% chance) in a 5‐year time frame (63, Figure 2b) compared to a 1‐year timeframe (17 of 433, Figure 2a). Relatively few counties (10 of 433) had a high probability (>60% chance) of having a year with at least 5 neuroinvasive cases within a 5‐year timeframe, and these were predominantly urban areas with high populations (Figure 2c). Two groups, containing 40 counties, never had a single reported case of WNV. For the remaining groups with WNV cases, counties with no observed cases were predicted to have a low probability of cases based on the group model (see Table S1 in Keyel, 2022a). Only one county (Washington, PA) had *a* <0.05 probability of having 20 years of no cases by chance assuming a similar risk to the rest of the counties in the group (Table S1 in Keyel, 2022a). When corrected for multiple comparisons, no county differed significantly from the negative binomial model (with 174 comparisons, ∼8–9 counties would be expected to have a *p* value < 0.05 by chance (Table S1 in Keyel, 2022a)).

**Figure 3:**
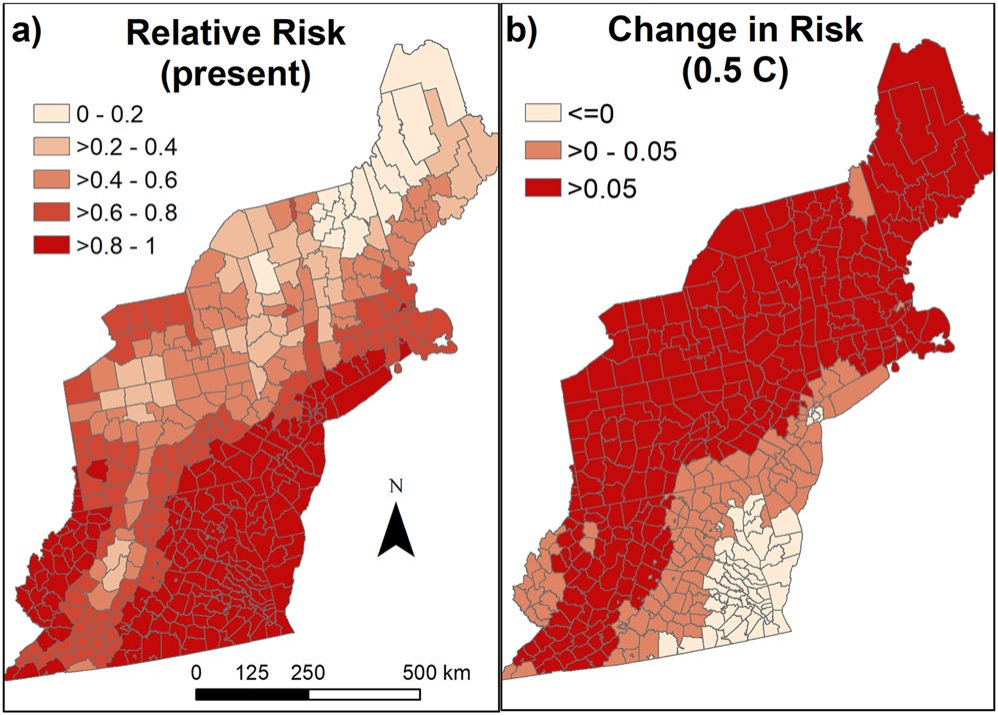
(a) Present‐day temperature‐trait‐predicted relative risk for *Culex pipiens*, and (b) the predicted change in temperature‐based risk with 0.5°C warming (on the high end for predicted for warming in the next decade). Note that this amount of warming falls within the range of present day temperature variation around the mean presented in (a). Map base layer from 2017 TIGER/Shapefiles (US Census Bureau, 2021).

When temperature‐based suitability was divided up into 5 equal intervals, 223 counties had the highest temperature‐based suitability for WNV, while 14 were in the lowest suitability category (Figure 3a). Most counties (84%, 365 of 433) were predicted to increase in temperature‐based suitability over the next decade (Figure 3b).

The five most densely populated urban areas are expected to remain in a suitable temperature range for WNV under future warming (Table 1). Once 0.5°C of warming has occurred, 4 of the 5 urban areas will decrease in temperature‐based suitability with further warming. However, substantial reductions in suitability for many major urban areas will not occur with less than 2.5°C of warming.

## 4 Discussion

A negative binomial distribution provides a simple method for describing patterns of WNV in the northeast (Keyel & Kilpatrick, 2021) and was among the top models in a national WNV forecasting challenge (Holcomb et al., 2023). The most important insight for public health is that a series of years with no or few WNV cases is possible even with a constant probability distribution for WNV cases. This means that reducing public health expenditures based on a few years with low WNV, on the assumption that it “has gone awayˮ is a poor strategy and will leave public health unprepared for the expected high years. WNV budgets should consider probabilities of WNV cases over at least 5‐year time horizons and have an emergency fund or the capacity to roll over funds from 1 year to the next, in order to address the expected high WNV years.

Further, areas with low rates of WNV may want to adopt a regional response approach that ensures counties have access to resources when cases occur. For most of the Northeast, a previous absence of a neuroinvasive WNV case over the past 20 years is not an indicator that the county will remain free of neuroinvasive WNV cases in the future, or even is lower risk than counties that have previously had cases. This suggests that these counties simply did not have cases due to low populations and random chance. The exceptions to this are in most of Maine (Figure 2) and southwestern Virginia, where no cases have been reported. Both of these areas are predicted to have increased temperature‐based suitability in the next decade due to climate change (Figure 3). This is likely to be more relevant for Maine than Virginia, as current temperature‐based suitability is relatively low in Maine. In Virginia, temperature‐based suitability is already high, suggesting some other factor is responsible for the reduced number of cases. Therefore, southwestern Virginia may not see an increase in number of cases due to warming.

In the long‐run, temperature‐based suitability for WNV is expected to increase across most of the Northeast, with the largest increases predicted in areas with relatively low present‐day suitability. Decreases in suitability are predicted for the southern portion of the region. Locations where WNV is relatively rare will need to be on the look‐out for an increase in cases (Figure 3b). These counties can expect to see substantial increases in temperature‐based suitability in the coming decades. Some of these regions should prepare to begin surveillance programs, doctors should familiarize themselves with WNV symptoms and lab work, and mosquito control operations should be prepared for expanded operations to reduce disease risk. That said, temperature‐based suitability is currently high in some localities that have low numbers of observed WNV neuroinvasive cases, and therefore other factors may also be critical in determining how numbers of WNV cases may change into the future. For example, land cover (Rochlin et al., 2008), mosquito control efforts (Bellini et al., 2014), mosquito microbiota (Novakova et al., 2017), population density (Rochlin et al., 2008), demographic structure of the human populations (Ruiz et al., 2004), socio‐economic status (Rothman et al., 2021), presence of septic systems (Myer et al., 2017), synchrony with avian breeding (Caillouët et al., 2013), among other factors influence WNV dynamics and could change WNV case numbers.

Locations with the most WNV cases in the present will have relatively little to do for long‐term climate‐change‐related planning for WNV. Existing mitigation measures should be as effective or more effective at controlling WNV in the future, as conditions shift to be less suitable for mosquito‐based transmission of WNV. This rests on the assumption of no evolution. However, temperature‐dependent evolution of the WNV has been shown in the lab (Fay et al., 2021) and in the field (Bialosuknia et al., 2022). A further caveat is this research was purely from the standpoint of WNV. In locations where WNV is expected to decline, other vector‐borne diseases, such as Zika virus, and the vectors that spread them, such as *Aedes aegypti*, may expand (Kraemer et al., 2019; Ryan et al., 2021). However, warming is not predicted to be sufficient to be suitable for dengue to become endemic in the Northeast under future climate change scenarios that extend out to 2080 (Messina et al., 2019).

The mismatch between probabilistic present‐day probabilities of human cases compared to temperature‐trait‐based suitability for the Northeast is interesting (compare Figures 2b and 3a). Virginia and West Virginia have lower probabilities of human cases than expected based on the temperature suitability models, while western and central Pennsylvania appears to have higher probabilities of cases than predicted by the temperature suitability models. Future work can explore whether landcover can explain these discrepancies, as prior research has suggested that urban areas are more favorable to WNV amplification (Bradley et al., 2008).

Another interesting future direction would be to compare the probabilities of human neuroinvasive cases with present‐day surveillance effort. Are some regions under‐surveyed for vector‐borne diseases? Could additional surveillance reduce human cases in these regions? Do some areas with high surveillance have fewer cases than predicted? Are they over‐surveyed, or does the enhanced surveillance lead to fewer cases?

## Conflict of Interest

The authors declare no conflicts of interest relevant to this study.

## Supporting information

Figure S1

## Data Availability

Neuroinvasive case data are available from the Centers for Disease Control Division of Vector‐borne Diseases upon request (dvbid2@cdc.gov), subject to a data use agreement. Maps were created using ArcGIS 10.6.1 (Redlands, CA) with 2017 US Census TIGER/Shapefiles as the county base layer (US Census Bureau, 2021). Population data were derived from US Census data (US Census Bureau, 2017, 2019). Code used to run the analysis is available from Zenodo (Keyel, 2022b). Data tables with expanded results are also available from Zenodo (Keyel, 2022a).
