# Supplementary material for: Patterns of West Nile Virus in the Northeastern United States Using Negative Binomial and Mechanistic Trait‐Based Models
Source: Geohealth. 2023 Apr 4;7(4):e2022GH000747. doi: 10.1029/2022GH000747 (PMC10072317; doi:10.1029/2022GH000747)
Supplement: Supplementary file 1 — Figure S1 [file GH2-7-e2022GH000747-s001.docx]

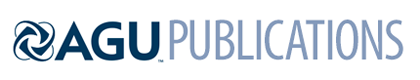


*GeoHealth*

Supporting Information for

**Patterns of West Nile virus in the Northeastern United States using negative binomial and mechanistic trait-based models**

Alexander C. Keyel^1,2^

1 Division of Infectious Diseases, Wadsworth Center, New York State Department of Health, Albany, NY, United States of America

2 Department of Atmospheric and Environmental Sciences, University at Albany, SUNY, Albany, NY, United States of America

**Contents of this file**

Figures S1 to Sx

**Introduction**

- A single supplemental figure to clarify the methods used in the manuscript


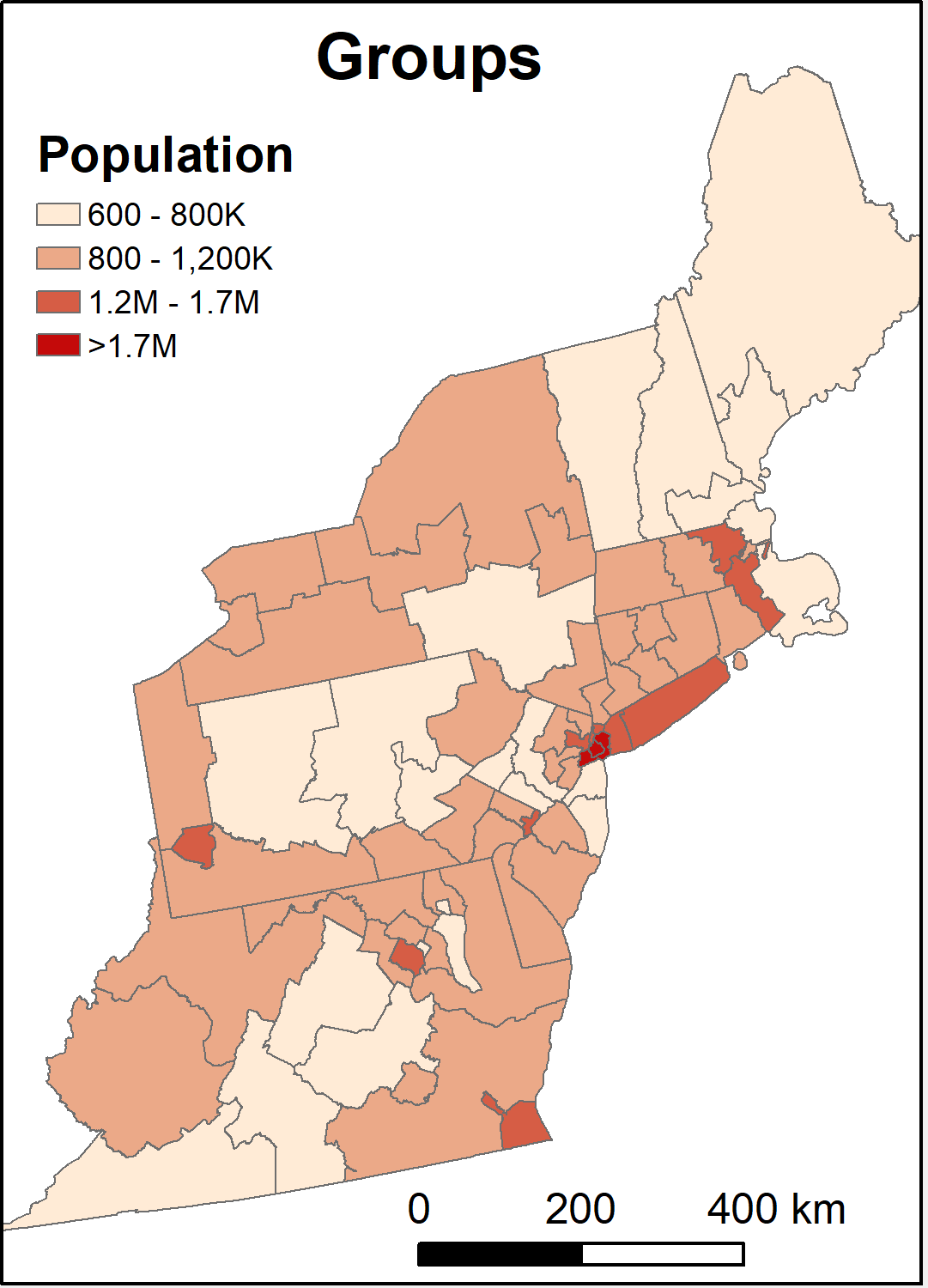


Figure S1. Map of population by groups used to fit the negative binomial model. Group assignment included a subjective element, and results for individual counties could vary depending on group assignment. Groups derived from merging 2017 TIGER/Shapefiles (US Census Bureau, 2021).
